# Associations between fast food and physical activity environments and adiposity in mid-life: cross-sectional, observational evidence from UK Biobank

**DOI:** 10.1016/S2468-2667(17)30212-8

**Published:** 2017-12-13

**Authors:** Kate E Mason, Neil Pearce, Steven Cummins

**Affiliations:** a Faculty of Epidemiology and Population Health, London School of Hygiene & Tropical Medicine, London, UK; b Department of Medical Statistics and Centre for Global Non-Communicable Diseases, London School of Hygiene & Tropical Medicine, London, UK; c Department of Social & Environmental Health Research, London School of Hygiene & Tropical Medicine, London, UK

## Abstract

**Background:**

The built environment might be associated with development of obesity and related disorders. We examined whether neighbourhood exposure to fast-food outlets and physical activity facilities were associated with adiposity in UK adults.

**Methods:**

We used cross-sectional observational data from UK Biobank. Participants were aged 40–70 years and attended 21 assessment centres between 2006 and 2010. Using linked data on environments around each participant's residential address, we examined whether density of physical activity facilities and proximity to fast-food outlets were associated with waist circumference, body-mass index (BMI), and body fat percentage. We used multilevel linear regression models adjusted for potential confounders, and conducted several sensitivity analyses.

**Findings:**

Complete case sample sizes were 401 917 (waist circumference models), 401 435 (BMI), and 395 640 (body fat percentage). Greater density of physical activity facilities within 1000 m of home was independently associated with smaller waist circumference and lower BMI and body fat percentage. Compared with people with no nearby facilities, those with at least six facilities close to home had 1·22 cm smaller waist circumference (95% CI −1·64 to −0·80), 0·57 kg/m^2^ lower BMI (−0·74 to −0·39), and 0·81 percentage points lower body fat (−1·03 to −0·59). Living further from a fast-food outlet was weakly associated with waist circumference and BMI, mostly among women. Compared with people living fewer than 500 m from a fast-food outlet, those living at least 2000 m away had 0·26 cm smaller waist circumference (−0·52 to 0·01).

**Interpretation:**

This study shows strong associations between high densities of physical activity facilities and lower adiposity for adults in mid-life. We observed weaker associations for access to fast food, but these are likely to be underestimated owing to limitations of the food environment measure. Policy makers should consider interventions aimed at tackling the obesogenic built environment.

**Funding:**

Commonwealth Scholarship Commission, Wellcome Trust Institutional Strategic Support Fund.

## Introduction

Obesity is strongly linked to a range of chronic diseases, including type 2 diabetes and cardiovascular disease, and contributes substantially to excess morbidity, mortality, and rising health-care costs globally.^1^, ^2^ Across the world, increasing urbanisation is now recognised as a key driver of obesity and related non-communicable diseases, prompting calls to improve understanding of how urban environmental factors influence health.^3^

Particularly in urban areas, features of neighbourhood environments, such as access to unhealthy food and few opportunities for physical activity, might be associated with the development of obesity and related disorders. Collectively, such features are often referred to as the obesogenic environment,^4^ and their presence and unequal distribution might partly explain rises in obesity prevalence and persistent social and geographical inequalities in obesity.^5^ Although much research has been done on the influence of various neighbourhood features on obesity, consistent evidence remains elusive.^4^ To further assess the potential of neighbourhoods to influence obesity outcomes via both energy intake and energy expenditure, and in particular the role of neighbourhood commercial resources, this Article focuses on two key features of the local residential environment: proximity to fast-food outlets and density of formal physical activity facilities. Recent studies indicate that about 21% of UK adults eat takeaway meals at home weekly^6^ and about 18% regularly use gyms, with smaller proportions participating in other forms of physical activity likely to involve formal facilities (eg, swimming and team or racquet sports).^7^

Previous research suggests that access to fast-food outlets near the home might be a determinant of weight status and obesity,^8^ although most supporting evidence comes from the USA, where the structure of the built environment differs from countries such as the UK. Although a recent study^9^ in Cambridgeshire, UK, found that exposure to fast-food outlets near home was associated with both body-mass index (BMI) and odds of obesity in adults, other international research has not consistently replicated findings from the USA.^10^, ^11^, ^12^

**Research in context**

**Evidence before this study**

We searched databases including PubMed, Web of Science, and Google Scholar, up to Aug 31, 2017, using various combinations of search terms including “neighbourhood”, “built environment”, “obesity”, “obesogenic”, “adiposity”, “food environment”, and “physical activity”, as well as hand-searching reference lists of relevant papers and tables of contents of recent or special editions of relevant journals. Our review of the literature was restricted to English language publications.

Much research has been done in recent decades into the effects of neighbourhood resources on obesity risk and related health behaviours. Features of the neighbourhood environment are hypothesised to affect diet quality through opportunities to consume healthy or unhealthy food (eg, access to fast food or grocery stores) and either encourage or discourage physical activity (eg, greenspace and street connectivity). However, the evidence base regarding so-called obesogenic environmental effects remains equivocal.

Many studies suggest that access to fast-food outlets in the local residential environment is a determinant of adiposity, but most evidence comes from the USA. Relatively little research has assessed the importance of local access to formal facilities for recreational physical activity (eg, gyms, leisure centres, swimming pools, and playing fields), instead focusing on broader urban design features such as walkability. In the UK, as elsewhere, many studies have been limited to a narrow geographical focus, relied on samples not sufficiently powered for subgroup analyses, or been prone to bias from residual confounding and exposure misclassification.

**Added value of this study**

This study is one of the first published to use UK Biobank to examine associations between features of the neighbourhood built environment and adiposity. The sample is made up of adults in mid-life—a crucial period of the lifecourse for the development of chronic disease. By using a very large dataset that covers much of the UK, we were able to provide evidence that relates to a wider geographical area than do most UK-based studies, and to examine sex and income differences. Making use of extensive covariate data available in UK Biobank, we were able to more comprehensively adjust for sources of confounding than have many other studies, and with additional sensitivity analyses, we were able to examine the robustness of our findings to residual confounding and different model specifications, further strengthening our findings.

We examined two features of the neighbourhood residential environment: proximity to fast-food outlets and density of formal physical activity facilities. We found evidence of a strong and graded inverse association between number of physical activity facilities close to home and three different adiposity measures. We observed a weaker association between the fast-food environment and adiposity, but limitations of the available food environment measure, such as misclassification of some fast-food outlets as restaurants and the inability to simultaneously account for both healthy and unhealthy food outlets, are likely to have attenuated the results. We also observed income and sex differences.

**Implications of all the available evidence**

The results of this study provide evidence to support the hypothesis that increasing access to local physical activity facilities and, possibly, reducing access to fast food close to residential areas has the potential to reduce overweight and obesity at the population level. Policy makers should consider interventions aimed at modifying residential environments to better facilitate healthy lifestyles, but recognising that such an approach might be more effective in some groups than in others. Future research should involve further interrogation of this rich data resource to examine geographical and other forms of heterogeneity in the effects of obesogenic environments on health to best target interventions.

A range of neighbourhood attributes have also been found to potentially influence physical activity;^13^ however, evidence of associations between obesity and neighbourhood attributes such as walkability and greenspace is mixed.^4^, ^14^ Relatively little research has assessed the importance of local access to formal facilities for recreational physical activity, such as gyms, swimming pools, and playing fields, although recent research in the UK indicates a possible relationship.^15^

Most studies in the UK have focused on particular geographical areas, such as individual cities or regions,^9^, ^11^, ^15^ with only one UK-wide study,^16^ which linked several neighbourhood-level contextual factors with obesity. Much like the international evidence, findings from the UK are mixed. This might be partly due to many studies being insufficiently powered, based on self-reported outcome measures, and focused on a narrow geographical area.

To improve understanding of how neighbourhood features influence adiposity and obesity-related outcomes in the UK, analyses of high-quality, individual, objective health data linked to environmental exposures measured at the individual address level are needed, for the whole of the UK. Using observational data from UK Biobank—a large sample of adults in a crucial period of the lifecourse for the development of chronic disease—we assessed whether the number of formal physical activity facilities near an individual's place of residence and proximity to fast-food outlets are independently associated with objectively measured adiposity. We also explore whether these associations differ by sex or income, and whether findings might be affected by residual confounding.

## Methods

### Study population

We used cross-sectional baseline data from UK Biobank (project 17380), a large, population-based cohort; the scientific rationale, study design, and survey methods for this project have been described elsewhere.^17^ Data were potentially available from 502 656 individuals who had visited the 22 UK Biobank assessment centres across the UK between 2006 and 2010. Individuals aged 40–69 years living within a 25-mile radius of an assessment centre and listed on National Health Service patient registers were invited to participate in the UK Biobank study. The age range was chosen by UK Biobank as an important period for the development of many chronic diseases. The final recruited sample was aged 37–73 years, with more than 99% of participants aged 40–69 years.

Linked to UK Biobank is a high-resolution spatial database of objectively measured characteristics of the physical environment surrounding each participant's exact residential address, derived from multiple national spatial datasets.^18^ The measures of the local environment include densities of various land uses, proximity to various health-relevant destinations (eg, general practitioners practices, industrial sites, fast-food outlets), greenspace, street-network accessibility, and pollution. The metrics were constructed using data collected in 2010, as close as possible to the baseline assessment of individuals.^18^

UK Biobank has ethics approval from the North West Multi-centre Research Ethics Committee (reference 16/NW/0274).

### Exposures

We defined the physical activity environment as the density (count) of formal physical activity facilities within a 1000 m street-network buffer around each individual's place of residence. Formal physical activity facilities were defined at address level as any land use classified in the Commercial-Leisure subcategory of the UK Ordnance Survey AddressBase Premium database.^18^ This subcategory comprises a range of indoor and outdoor facilities designed for sporting and leisure activities, such as gyms, swimming pools, and playing fields (see appendix for details). We did not include informal physical activity facilities, such as public parks and cycling paths (except where covered by the above classification—eg, playing fields), because formal physical activity facilities are understudied as a neighbourhood health resource and might have different drivers of use. Because many of these facilities are commercial, they are also potentially modifiable via regulatory and commercial levers that are less relevant to the informal physical activity environment.

For each individual, the street-network distance (in metres) from residential address to the nearest fast-food outlet, classified as “hot/cold fast-food outlet/takeaway” in the UK Ordnance Survey AddressBase Premium database,^18^ was available. We used these distances and the distribution of data to categorise individuals as living closer than 500 m, 500–999 m, 1000–1999 m, or at least 2000 m from their nearest fast-food outlet. A proximity measure was used by contrast with the physical activity environment density measure because no equivalent density measure was available for the food environment. Density and proximity measures in the UK have been shown to be correlated despite being theoretically distinct.^19^

### Adiposity measures

We used three adiposity measures: waist circumference, BMI (calculated from height and weight), and body fat percentage (measured by bioimpedance). Measurements were made by trained staff using standard procedures.^17^ We centred all three metrics around their mean and treated them as continuous variables.

### Potential confounders and model adjustments

We identified potential confounders for each of the two environmental exposures on the basis of the existing literature, and summarised this information in directed acyclic graphs to assess which should be adjusted for in the main analyses (appendix). Potential confounders included individual demographic and socioeconomic variables and several local area characteristics. Because the fast-food and physical activity environments are also associated with one another, features of the food environment might confound associations between the physical activity environment and adiposity, and the physical activity environment might confound associations between the food environment and adiposity. Although individual diet and physical activity behaviours are associated with adiposity, physical activity is unlikely to be a common ancestor of the food environment and adiposity, and similarly diet will not predict the physical activity environment. Furthermore, conditioning on these behaviours risks inducing collider bias^20^ by opening backdoor pathways through genetic risk, prior behaviours, and prior adiposity.

On the basis of these causal diagrams, we adjusted the final models for age (years), sex (male or female), ethnicity (white, south Asian, black, other Asian, mixed white and black, mixed white and Asian, mixed other, or other), highest education level attained (college or university degree; A levels, AS levels, or equivalent [academic advanced levels, post compulsory education]; O levels, GCSEs, or equivalent [higher secondary education]; CSE or equivalent [secondary education]; National Vocational Qualification, Higher National Diploma, Higher National Certificate, or equivalent [vocational qualifications]; other professional qualification; or none of the above), annual household income (<£18 000, £18 000–30 999, £31 000–51 999, £52 000–100 000, or >£100 000), employment status (paid work, retired, unable to work, unemployed, or other), area deprivation (Townsend score), urbanicity (urban or non-urban), and neighbourhood residential density (count of residential dwellings within a 1000 m street-network buffer of home address, log transformed). We adjusted models of the physical activity environment and adiposity for fast-food proximity, and adjusted models of the fast-food environment and adiposity for density of physical activity facilities.

We used negative control analyses to analyse possible residual confounding. Negative control analysis is a technique that can help to detect residual confounding in observational studies.^21^ Because studies of neighbourhoods and health are potentially susceptible to residual confounding by factors such as other neighbourhood characteristics and residential segregation, we employed this technique by doing analyses similar to our primary analyses, but in which the outcome was a variable not expected to be associated with our exposures; specifically, we used height (cm) as a negative control outcome because height is unlikely to be related to neighbourhood environment in adulthood, but, as with adiposity, it is correlated with various sociodemographic characteristics of individuals. If the main associations for adiposity were residually confounded, we would expect to observe a spurious association between the environmental exposures and height.

### Statistical analysis

We used multilevel, multiple linear regression models with random intercepts and random coefficients for the main exposure to estimate independent associations between each environmental exposure and each adiposity outcome, accounting for the nesting of individuals within assessment centres. We initially adjusted only for age and sex (model 0), then for likely demographic confounders (age, sex, ethnicity, area deprivation, and urbanicity; model 1), then further adjusted for individual-level socioeconomic characteristics (income, education, and employment status; model 2) and, finally, for the non-exposure environmental feature (proximity to fast food or density of physical activity facilities) and neighbourhood residential density (model 3). As well as adjusting for potential confounding by sex and income, we also tested fully adjusted models for effect modification by these variables. We report stratified results where models with interaction terms for sex or income were statistically different from those without (likelihood ratio test p<0·05). Finally, we estimated the same models using height as a negative control outcome.

We designed sensitivity analyses to test the robustness of our findings to different model specifications. These analyses examined the effect of further adjustment for the behavioural variables diet and physical activity; the extent to which any such effect was driven by selection bias due to missing data; and the sensitivity of our models to the choice of BMI measure.

We did all analyses in Stata SE version 14.2.

### Role of the funding source

The funder had no role in the design, conduct, or writing up of this study. The corresponding author had full access to all of the data and final responsibility to submit for publication.

## Results

Environmental data were available for individuals from 21 of the 22 assessment areas; no environmental data were collected for participants assessed in Stockport, where the pilot study was conducted. Of the primary covariates (excluding diet and physical activity), income had the most missing data, followed by residential density, BMI, and body fat percentage (table 1). All other covariates were missing at a frequency of approximately 1% or less (table 1). After the exclusion of cases missing data on key covariates, the final complete case sample sizes were 401 917 for waist circumference analyses, 401 435 for BMI analyses, and 395 640 for body fat analyses. Excluded observations were very similar to complete cases in terms of neighbourhood exposures and adiposity, but were more often people from more deprived postcodes, less highly educated, more likely to be retired, and more likely to be of south Asian or black ethnicity.

**Table 1:** Characteristics of study participants

			**Data (n=498 822)**
**Adiposity**			
Waist circumference, cm			90·3 (13·5)
	Range		20 to 197
	Data missing		2110 (0·4%)
Body-mass index, kg/m^2^			27·4 (4·8)
	Range		13 to 68
	Data missing		10 014 (2·0%)
Body fat			31·5% (8·5)
	Range		5% to 70%
	Data missing		10 286 (2·1%)
**Environment**			
Physical activity environment			
	Number of facilities in 1000 m buffer		1 (0 to 3)
		0	150 211 (31·2%)
		1	96 031 (20·0%)
		2 to 3	114 693 (23·8%)
		4 to 5	58 217 (12·1%)
		≥6	61 978 (12·9%)
		Range	0 to 39
	Data missing		17 692 (3·5%)
Fast-food environment			
	Distance to nearest outlet, m		1136 (615 to 2197)
		<500 m	88 804 (18·5%)
		500 m to 999 m	124 698 (25·9%)
		1000 m to 1999 m	133 401 (27·7%)
		≥2000 m	134 180 (27·9%)
		Range	0 to 96 538
	Data missing		17 719 (3·6%)
**Covariates**			
Age, years*			56·5 (8·1)
	Range		40 to 70
	Data missing		15 (0·0%)
Sex			
	Female		271 384 (54·4%)
	Male		227 438 (45·6%)
	Data missing		1 (0·0%)
Ethnicity			
	White		469 209 (94·6%)
	South Asian or south Asian British		8015 (1·6%)
	Black or black British		8038 (1·6%)
	Chinese or other (non-South) Asian		3367 (0·7%)
	Mixed: white and black		1029 (0·2%)
	Mixed: white and Asian		827 (0·2%)
	Mixed: detail unknown		1073 (0·2%)
	Other		4521 (0·9%)
	Data missing		2743 (0·5%)
Income			
	Less than £18 000		97 221 (22·9%)
	£18 000–30 999		108 197 (25·4%)
	£31 000–51 999		110 790 (26·0%)
	£52 000–100 000		86 280 (20·3%)
	Greater than £100 000		22 933 (5·4%)
	Data missing		73 401 (14·7%)
Education†			
	College or university degree		161 200 (32·7%)
	A levels, AS levels, or equivalent		55 331 (11·2%)
	O levels, GCSEs, or equivalent		105 218 (21·4%)
	CSE or equivalent		26 893 (5·5%)
	NVQ, HND, HNC, or equivalent		32 734 (6·6%)
	Other professional qualifications		25 810 (5·2%)
	None of the above		85 291 (17·3%)
	Data missing		6345 (1·3%)
Employment status			
	Paid employment or self-employed		284 873 (57·4%)
	Retired		165 977 (33·5%)
	Unable to work		16 654 (3·4%)
	Unemployed		8225 (1·7%)
	Home duties, carer, student, volunteer, or other		20 171 (4·1%)
	Data missing		2922 (0·6%)
Area deprivation (Townsend index)			−2·1 (−3·6 to 0·5)
Data missing			626 (0·1%)
Urbanicity			
	Urban		425 082 (86·1%)
	Non-urban		68 665 (13·9%)
	Data missing		5075 (1·0%)
Residential density‡			1899 (1095 to 3102)
	Range		1 to 22 306
	Data missing		17 705 (3·5%)
Total dietary energy intake, kJ			8752·6 (2784·6)
	Range		0 to 19 995
	Data missing		288 690 (57·9%)
Physical activity (MET min per week)			1666 (743 to 3413)
	Range		0 to 32 130
	Data missing		43 656 (8·8%)
**Assessment area**			
Manchester			13 940 (2·8%)
Oxford			14 062 (2·8%)
Cardiff			17 882 (3·6%)
Glasgow			18 651 (3·7%)
Edinburgh			17 201 (3·4%)
Stoke			19 440 (3·9%)
Reading			29 417 (5·9%)
Bury			28 335 (5·7%)
Newcastle			37 008 (7·4%)
Leeds			44 209 (8·9%)
Bristol			43 015 (8·6%)
Central London			12 583 (2·5%)
Nottingham			33 877 (6·8%)
Sheffield			30 397 (6·1%)
Liverpool			32 818 (6·6%)
Middlesbrough			21 289 (4·3%)
Hounslow			28 879 (5·8%)
Croydon			27 385 (5·5%)
Birmingham			25 503 (5·1%)
Swansea			2281 (0·5%)
Wrexham			649 (0·1%)

Summary statistics were examined for the full sample of participants from the 21 areas linked to the environmental dataset. Data are n (%), mean (SD), or median (IQR), and are given for the complete case sample for each category, with number and percentage of records with missing data displayed for all variables with missing entries. Due to rounding error, some percentages sum to more than 100%. MET=metabolic equivalent of task.

* Our analytical sample included people aged 70 years at the time of assessment, but excluded nine individuals with complete data who were younger than 40 years or older than 70 years.

† UK qualifications break down into academic advanced levels (A levels, AS levels, or equivalent, post compulsory education), higher secondary education (O levels, GCSEs, or equivalent), secondary education (CSE or equivalent), and vocational qualifications (NVQ, HND, HNC, or equivalent).

‡ Residential address points per 1000 m buffer.

The mean waist circumference of the full sample with environmental data available was 90·3 cm, mean BMI was 27·4 kg/m^2^, and mean body fat percentage was 31·5% (table 1). The median number of formal physical activity facilities within a 1000 m street-network distance of participants' homes was one, with a third of participants having no facilities close to home (table 1). Participants lived a median of 1136 m from a fast-food outlet, with nearly a fifth living within 500 m of such an outlet (table 1).

In fully adjusted models, adiposity was lower among people with greater access to local physical activity facilities compared with those with fewer facilities near home (table 2). Compared with the reference category (no nearby physical activity facilities), the waist circumference of those with at least six facilities nearby was, on average, 1·22 cm smaller (95% CI −1·64 to −0·80; p<0·0001); their BMI was 0·57 kg/m^2^ lower (−0·74 to −0·39; p<0·0001); and their body fat was 0·81 percentage points lower (−1·03 to −0·59; p<0·0001). Regression coefficients decreased monotonically across categories of increasing density.

**Table 2:** Associations between density of physical activity facilities and adiposity outcomes: multilevel regression results

		**Model 0**	**Model 1**	**Model 2**	**Model 3**
**Waist circumference, cm (n=401 917)**					
Number of facilities					
	0	0 (ref)	0 (ref)	0 (ref)	0 (ref)
	1	0·15 (−0·02 to 0·32)	−0·15 (−0·30 to −0·01)	−0·13 (−0·26 to 0·00)	−0·19 (−0·32 to −0·06)
	2–3	0·08 (−0·20 to 0·35)	−0·43 (−0·70 to −0·17)	−0·29 (−0·51 to −0·08)	−0·40 (−0·62 to −0·17)
	4–5	−0·14 (−0·55 to 0·27)	−0·80 (−1·19 to −0·42)	−0·51 (−0·82 to −0·19)	−0·65 (−0·96 to −0·33)
	≥6	−0·67 (−1·25 to −0·09)	−1·51 (−2·04 to −0·98)	−1·03 (−1·45 to −0·62)	−1·22 (−1·64 to −0·80)
**Body-mass index, kg/m^2^ (n=401 435)**					
Number of facilities					
	0	0 (ref)	0 (ref)	0 (ref)	0 (ref)
	1	0·04 (−0·04 to 0·12)	−0·08 (−0·15 to 0·00)	−0·06 (−0·13 to 0·01)	−0·07 (−0·14 to 0·00)
	2–3	−0·03 (−0·15 to 0·10)	−0·22 (−0·34 to −0·09)	−0·16 (−0·26 to −0·05)	−0·18 (−0·28 to −0·08)
	4–5	−0·18 (−0·36 to 0·00)	−0·43 (−0·59 to −0·26)	−0·30 (−0·43 to −0·17)	−0·33 (−0·46 to −0·20)
	≥6	−0·42 (−0·67 to −0·17)	−0·73 (−0·96 to −0·50)	−0·52 (−0·69 to −0·34)	−0·57 (−0·74 to −0·39)
**Body fat, % (n=395 640)**					
Number of facilities					
	0	0 (ref)	0 (ref)	0 (ref)	0 (ref)
	1	0·03 (−0·07 to 0·14)	−0·11 (−0·20 to −0·01)	−0·08 (−0·16 to 0·00)	−0·11 (−0·20 to −0·03)
	2–3	−0·07 (−0·24 to 0·11)	−0·30 (−0·46 to −0·13)	−0·21 (−0·34 to −0·07)	−0·27 (−0·40 to −0·13)
	4–5	−0·28 (−0·53 to −0·02)	−0·58 (−0·81 to −0·35)	−0·40 (−0·58 to −0·22)	−0·48 (−0·67 to −0·29)
	≥6	−0·63 (−0·96 to −0·30)	−1·00 (−1·29 to −0·70)	−0·71 (−0·92 to −0·49)	−0·81 (−1·03 to −0·59)

Density is defined as number of physical activity facilities in a 1000 m street-network buffer. Data are mean difference (95% CI). Model 0: adjusted for age and sex. Model 1: model 0 plus adjustment for ethnicity, urban or non-urban status, and area deprivation. Model 2: model 1 plus adjustment for individual socioeconomic characteristics (income, education, and employment status). Model 3: model 2 plus adjustment for residential density and distance to nearest fast-food outlet.

Main effect estimates were smallest in models adjusted only for age and sex (model 0; table 2). In fully adjusted models (model 3), physical activity environment coefficients were attenuated compared with models that also controlled for ethnicity, urbanicity, and area deprivation (model 1), but were larger in magnitude than intermediate models further adjusted for individual socioeconomic characteristics (model 2; table 2).

**Figure 1:**
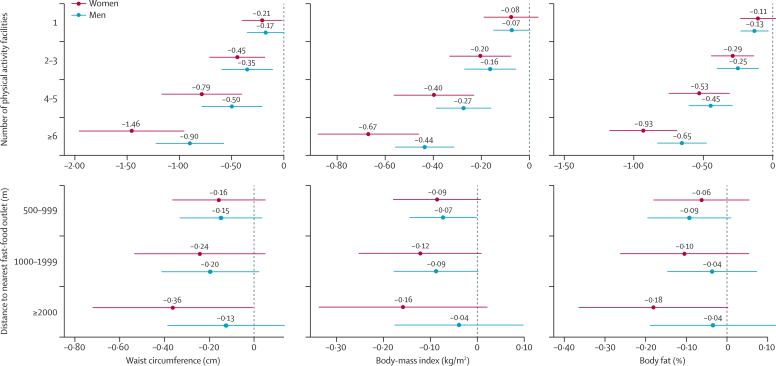
Association between neighbourhood environmental features and adiposity, by sex Figure shows sex-stratified, fully adjusted mean differences in adiposity and associated 95% CIs. The dashed line at zero represents the reference category (no physical activity facilities with 1000 m of home or <500 m to nearest fast-food outlet).

**Figure 2:**
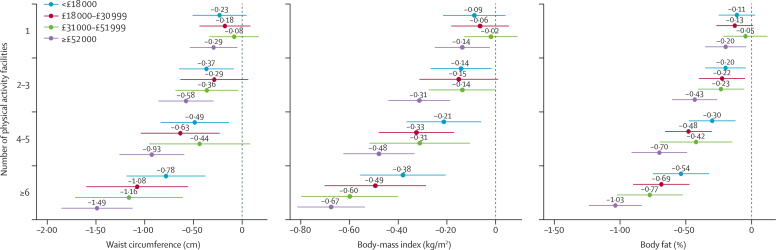
Association between number of formal physical activity facilities and adiposity, by annual household income Figure shows annual-household-income-stratified, fully adjusted mean differences in adiposity and associated 95% CIs. The dashed line at zero represents the reference category (no physical activity facilities with 1000 m of home). Income-stratified results for the association between distance to nearest fast-food outlet and adiposity can be found in the appendix.

For the associations between physical activity facilities and adiposity, we found evidence of effect modification by sex (waist circumference p<0·0001; BMI p=0·0009; body fat p=0·0029) and income (waist circumference and BMI both p<0·0001; body fat p=0·0026), with an inverse association for all subgroups but stronger among women (figure 1) and people from higher-income households (figure 2).

We also estimated lower mean waist circumference with each categorical increase in distance to the nearest fast-food outlet, independent of the influence of the physical activity environment and all other covariates (ie, model 3; table 3). However, the only significant coefficients estimated were for intermediate proximity categories, and these were small: compared with participants living within 500 m of a fast-food outlet, the mean waist circumference among those living within 500–999 m was 0·15 cm smaller (95% CI −0·30 to −0·01, p=0·040) and 0·22 cm smaller (−0·44 to 0·00, p=0·049) for those living within 500–1499 m (table 3). For those living at least 2000 m from an outlet, the average decrease was 0·26 cm (95% CI −0·52 to 0·01; p=0·057). We observed a similar pattern of association for BMI. For body fat percentage, evidence of an association was weaker—eg, for people living at least 2000 m from a fast-food outlet, body fat was 0·10 percentage points lower (p=0·18). Sex modified these relationships for all three outcome measures (p<0·0001 for all measures; figure 1), whereas income did not (waist circumference p=0·094; BMI p=0·423; body fat p=0·083; appendix). With regard to sex, only among women did we observe an inverse dose–response association, with lower average adiposity across all measures the further a woman lived from a fast-food outlet, although only the waist circumference coefficient for the greatest distance category (≥2000 m) reached significance at the 5% level (0·36 cm decrease, 95% CI −0·72 to −0·01). We observed no such association for men.

**Table 3:** Associations between proximity to fast-food outlets and adiposity outcomes: multilevel regression results

		**Model 0**	**Model 1**	**Model 2**	**Model 3**
**Waist circumference, cm (n=401 917)**					
Distance					
	<500 m	0 (ref)	0 (ref)	0 (ref)	0 (ref)
	500–999 m	−0·48 (−0·67 to −0·29)	−0·08 (−0·24 to 0·07)	−0·07 (−0·22 to 0·07)	−0·15 (−0·30 to −0·01)
	1000–1999 m	−0·69 (−1·01 to −0·38)	−0·04 (−0·29 to 0·22)	−0·08 (−0·27 to 0·12)	−0·22 (−0·44 to 0·00)
	≥2000 m	−1·20 (−1·59 to −0·81)	−0·05 (−0·40 to 0·30)	−0·11 (−0·36 to 0·14)	−0·26 (−0·52 to 0·01)
**Body-mass index, kg/m^2^ (n=401 435)**					
Distance					
	<500 m	0 (ref)	0 (ref)	0 (ref)	0 (ref)
	500–999 m	−0·20 (−0·29 to −0·11)	−0·04 (−0·10 to 0·03)	−0·03 (−0·09 to 0·03)	−0·08 (−0·14 to −0·02)
	1000–1999 m	−0·24 (−0·39 to −0·09)	0·01 (−0·11 to 0·12)	−0·01 (−0·10 to 0·08)	−0·10 (−0·20 to −0·01)
	≥2000 m	−0·40 (−0·60 to −0·21)	0·03 (−0·15 to 0·22)	0·01 (−0·13 to 0·14)	−0·10 (−0·24 to 0·04)
**Body fat, % (n=395 640)**					
Distance					
	<500 m	0 (ref)	0 (ref)	0 (ref)	0 (ref)
	500–999 m	−0·18 (−0·27 to −0·08)	−0·02 (−0·10 to 0·07)	−0·02 (−0·10 to 0·06)	−0·08 (−0·16 to 0·00)
	1000–1999 m	−0·19 (−0·35 to −0·03)	0·07 (−0·07 to 0·21)	0·04 (−0·07 to 0·15)	−0·07 (−0·19 to 0·05)
	≥2000 m	−0·46 (−0·67 to −0·24)	0·05 (−0·15 to 0·26)	0·00 (−0·14 to 0·15)	−0·10 (−0·26 to 0·05)

Distances given are distance to nearest fast-food outlet. Data are mean difference (95% CI). Model 0: adjusted for age and sex. Model 1: model 0 plus adjustment for ethnicity, urban or non-urban status, and area deprivation. Model 2: model 1 plus adjustment for individual socioeconomic characteristics (income, education, and employment status). Model 3: model 2 plus adjustment for residential density and density of local physical activity facilities.

Fast-food-environment coefficients in fully adjusted models were substantially attenuated compared with models adjusted only for age and sex, but were larger in magnitude than in both intermediate models, suggesting that failure to adjust for neighbourhood-level confounders attenuates results.

The negative control analysis, using height as an outcome, yielded the expected null results for the fast-food environment, but showed evidence of increasing height with an increasing number of formal physical activity facilities; regression coefficients were small in magnitude (all <0·52 cm difference) but significant for the subgroups of 2–3 facilities, 4–5 facilities, and six facilities or more (table 4), suggesting some possible residual confounding.

**Table 4:** Results from negative control analyses

		**Height, cm (n=401 676)**
Number of physical activity facilities in 1000 m street-network buffer*		
	0	1 (ref)
	1	0·09 (0·01 to 0·16)
	2–3	0·21 (0·10 to 0·32)
	4–5	0·38 (0·27 to 0·49)
	≥6	0·51 (0·36 to 0·66)
Distance to nearest fast-food outlet†		
	<500 m	1 (ref)
	500–999 m	0·01 (−0·06 to 0·09)
	1000–1999 m	−0·02 (−0·11 to 0·08)
	≥2000 m	0·01 (−0·10 to 0·13)

Adjusted for age, sex, ethnicity, urban or non-urban status, area deprivation, income, education, employment status, and residential density.

* Also adjusted for distance to nearest fast-food outlet.

† Also adjusted for density of local physical activity facilities.

Additional adjustment for behavioural confounders had negligible impact on the regression coefficients (appendix). Adjustment for diet in a sensitivity analysis of physical activity models did not lead to substantively different conclusions but did inflate some point estimates; however, sensitivity analyses that estimated model 3 on a sample restricted to people with dietary data produced almost identical results, indicating that the observed inflation is driven by sample restriction due to missing data rather than solely additional adjustment for diet (appendix). Models that excluded the small proportion of BMI values measured manually rather than with the bioimpedance machine yielded substantively identical results to those from the primary analysis (appendix).

## Discussion

In this uniquely large and geographically diverse sample of adults in mid-life, we found consistent evidence that local access to formal physical activity facilities such as leisure centres, gyms, and sports fields is independently associated with adiposity. As the density of formal physical activity facilities increased, waist circumference, BMI, and body fat percentage all decreased. We observed a similar but much weaker association between proximity to fast-food outlets and adiposity. This dataset provided a unique opportunity to examine these associations in a sample spanning diverse areas of the UK rather than being limited to a single study site.

Physical activity facilities in the local environment provide convenient opportunities for recreational physical activity. If improved access increases physical activity behaviour, we would expect to see a causal effect on adiposity. In income-stratified models, the inverse association we observed for all income groups was most marked among higher-income households. This finding is unsurprising given many facilities have costs attached to use, and has implications for municipal and private providers of physical activity facilities, who should be encouraged to invest in facilities in or near residential areas, but also to ensure that costs of access are managed to avoid inadvertently widening health inequalities.

Although our findings regarding physical activity facilities are consistent with a recent Scottish study^15^ that found some measures of accessibility of physical activity facilities to be associated with BMI, our food environment findings are somewhat inconsistent with a recent study^9^ in England that found a strong, graded association between fast-food access and BMI, whereas we observed only a weak association. Food outlet classification in the source database for the current study is supplied by local authorities and might include misclassification of some outlets as restaurants rather than fast-food outlets, potentially biasing our regression coefficients towards the null. Given the limits of the available data, we were also unable to account for other dimensions of the food environment, which might have biased our estimates (see appendix p 2). Indeed, empirical evidence from other studies suggests that simultaneously accounting for healthy and unhealthy food outlets yields larger and more precise estimates of health effects than when considering only a single dimension of the food environment.^9^, ^22^, ^23^ This possibility of bias is further supported indirectly by the observation from our intermediate models that main effects were attenuated when no adjustment was made for other area-level confounders. There might also be local variability in the accuracy and completeness of these data, which we have not been able to assess. Stronger measures of the food environment linked to this dataset would provide more robust evidence on which to make more reliable inferences about the role of the fast-food environment in the UK. For many people, environmental determinants of diet and weight are also likely to include commuting routes and workplaces,^24^ but we were unable to assess these. Associations between adiposity and neighbourhood food environments might also not be consistent across geographical regions, even within the same country.^25^, ^26^

We found strong evidence of effect modification by sex, and stratified models showed modestly larger estimates of effects of both neighbourhood exposures on women's adiposity than on men's. Other studies have also observed sex differences in neighbourhood effects, in which women in some age groups appeared more sensitive to health impacts of local environments.^27^, ^28^ These differences might relate to traditional gender roles that result in women spending more time than men in their local neighbourhood, although this has not been clearly established.^28^

The negative control analysis for the physical activity environment indicates possible residual confounding, because we observed an unexpected association with height. Comparison of standardised coefficients across the relevant models (not shown) suggests this residual confounding would only partially account for the observed effect. For the physical activity environment, we observed in intermediate models that failure to adjust for individual socioeconomic characteristics biased coefficients away from the null. Therefore, if individual socioeconomic position has not been adequately controlled for (eg, insufficient specificity in the categorical, non-equivalised income variable), main effects might be overestimated.

This study has several strengths. The unique scale and size of UK Biobank reflect a geographical coverage that no other similar studies have, particularly in the UK, and enables examination of subgroup heterogeneity often not possible in smaller studies. We investigated two neighbourhood exposures, based on exact home address, thought to influence the same outcome via separate pathways, and assessed their independent associations with adiposity by controlling one for the other. Both exposures are primarily commercial in nature, and therefore amenable to regulatory and market-based interventions. One of these exposures—formal physical activity facilities—has received relatively little research attention to date. We examined associations with multiple objective biomarkers of adiposity because available measures vary in how well they predict different health outcomes in different populations.^29^ The consistency we observed across multiple outcome measures, especially for the physical activity environment, can be seen as providing stronger evidence of a potentially causal relationship. Sensitivity analyses suggest our main findings are robust to model misspecification, and that adjustment for health behaviours is not necessary, and in some cases might induce bias. Our findings were also consistent in preliminary analyses using alternative cutpoints for categories of the exposure variables (not shown). Although modest, the adiposity differences observed are averages across the sample, including people unlikely to eat fast food or use physical activity facilities regardless of local accessibility. If these observed differences do represent a causal relationship, the actual magnitude of effect would be larger among those likely to be affected.

In addition to weaknesses of the food environment measure highlighted above, the study has some further limitations. Although the UK Biobank sample is very large, the response rate was low (5·5%) and the sample shows evidence of so-called healthy volunteer bias.^30^ The non-null associations we observed between access to physical activity facilities and height in a negative control analysis suggest that some unmeasured confounding of the physical activity environment associations with adiposity remained. Studies of neighbourhood effects are particularly susceptible to bias arising from residential mobility, where movement between neighbourhoods over time increases the risk of exposure misclassification, and leaves open the potential for reverse causation in cross-sectional analyses if, for example, individuals with lower adiposity choose to live in areas with more physical activity facilities. Current adiposity might also reflect exposure to neighbourhood environments earlier in life, posing a further challenge for causal inference. Therefore, an important strength of this sample is its stability: more than 65% of respondents had been resident at their current address for at least 10 years (mean 17·3 years [SD 11·8]). Despite this, some risk of exposure misclassification might remain owing to retailer turnover, especially for fast-food outlets. Furthermore, the relationship between the fast-food environment and adiposity is likely to be bidirectional, but within this study we were unable to disentangle the possible effects of retailers positioning fast-food outlets in areas of higher demand. Another limitation of our analyses is the unavailability of equivalent metrics for the physical activity and food environments that might allow a more direct comparison of their influence.

Research on neighbourhood environments and obesity has produced inconsistent findings to date. Our findings from a large and geographically diverse sample of adults in mid-life add support to the hypothesis that increasing access to physical activity facilities and, possibly, reducing access to fast food close to residential areas has the potential to reduce the prevalence of obesity and overweight at the population level, but highlight that this approach might be more effective for some groups than for others.
