# Supplementary material for: Associations between fast food and physical activity environments and adiposity in mid-life: cross-sectional, observational evidence from UK Biobank
Source: Lancet Public Health. 2017 Dec 13;3(1):e24–33. doi: 10.1016/S2468-2667(17)30212-8 (PMC5764749; doi:10.1016/S2468-2667(17)30212-8)
Supplement: Supplementary appendix [file mmc1.pdf]

THE LANCET
Public Health

Supplementary appendix

This appendix formed part of the original submission and has been peer reviewed. We post it as supplied by the authors.

Supplement to: Mason KE, Pearce N, Cummins S. Associations between fast food and physical activity environments and adiposity in mid-life: cross-sectional, observational evidence from UK Biobank. *Lancet Public Health* 2017; published online Dec 12. http://dx.doi.org/10.1016/S2468-2667(17)30212-8.

SUPPLEMENTARY MATERIAL

1. Directed Acyclic Graphs for Relationships between Neighbourhood Environments (fast-food proximity and density of local formal PA facilities) and Adiposity

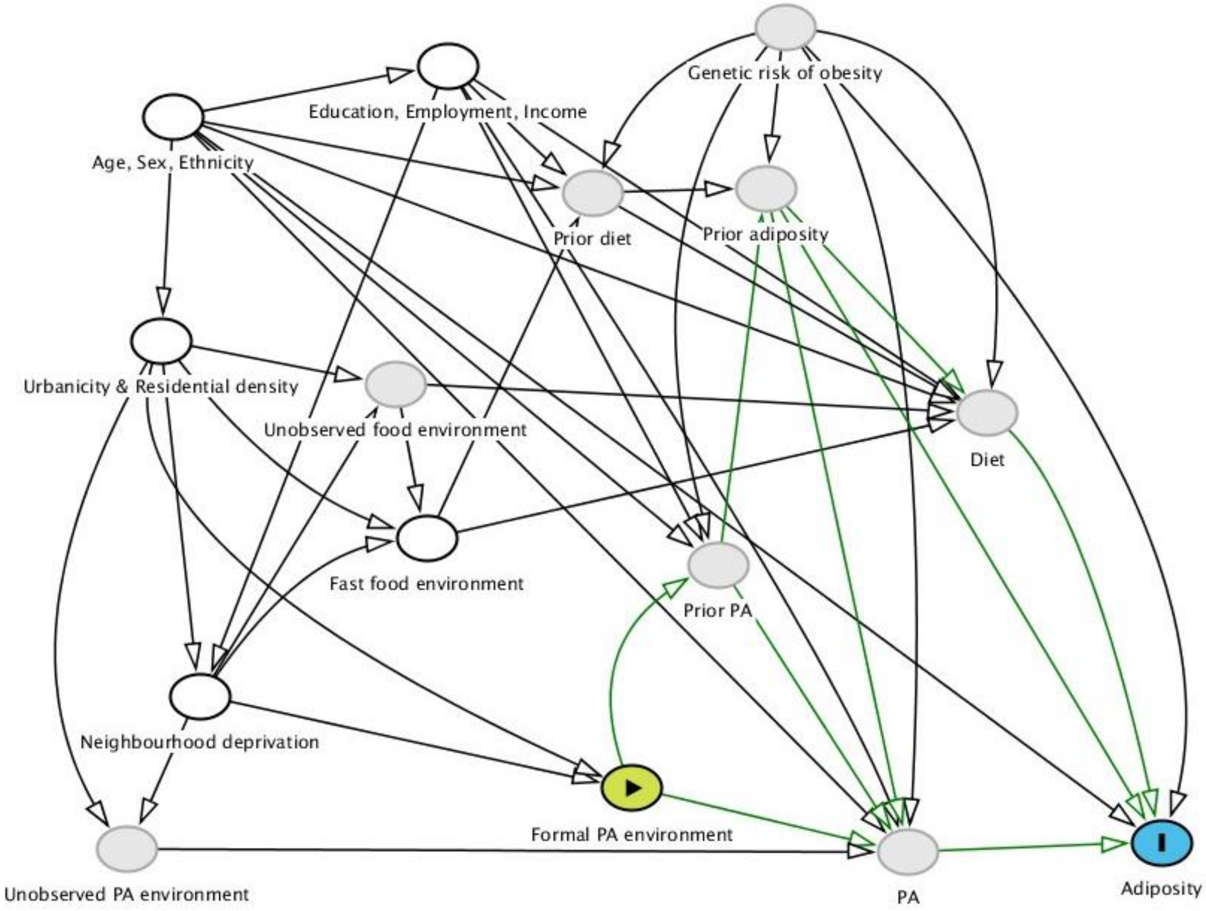

Supplementary Figure 1a. Formal physical activity environment – adiposity DAG

Yellow = exposure Blue = outcome White = adjusted potential confounders
Grey = unadjusted or unobserved potential confounders Green line = causal pathway
Red line = potential biasing pathway (absence of red line indicates that adjustment for minimal sufficient set of confounders is achieved)

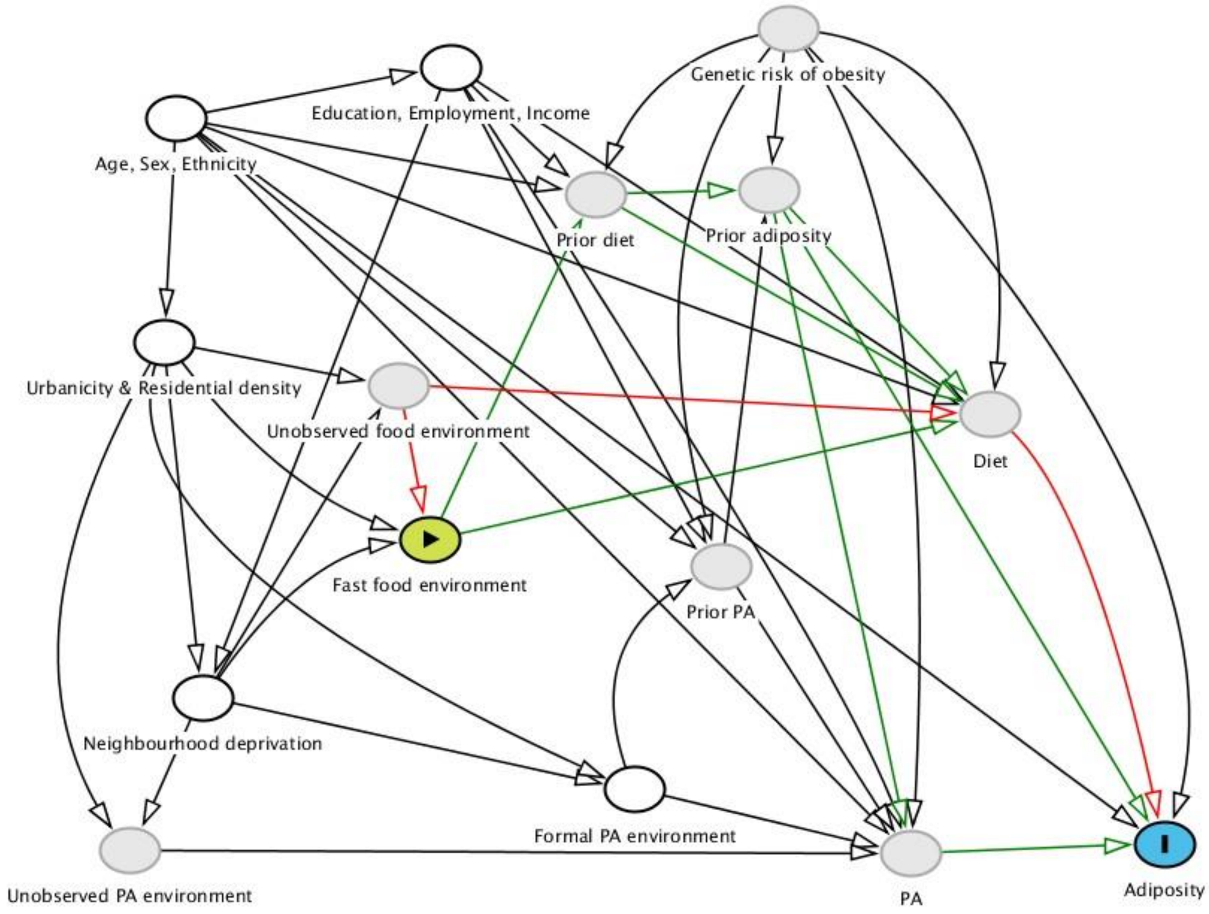

Supplementary Figure 1b. Fast food environment – adiposity DAG

Yellow = exposure Blue = outcome White = adjusted potential confounders
Grey = unadjusted or unobserved potential confounders Green line = causal pathway
Red = potential biasing pathway

2. Classification of Formal Physical Activity Facilities

Formal PA facilities were defined as any land use classified in the Commercial-Leisure subcategory (CL06) of the UK Ordnance Survey AddressBase Premium database (https://www.ordnancesurvey.co.uk/business-and-government/help-and-support/products/addressbase-premium.html). The data are contributed by local authorities, and covers municipal and private facilities for all sporting activities. This subcategory comprises any Indoor/Outdoor Leisure/Sporting Activity/Centre not further defined, as well as the following more specific categories of land use:

- Bowls Facility
- Cricket Facility
- Diving / Swimming Facility
- Equestrian Sports Facility
- Football Facility
- Golf Facility
- Activity / Leisure / Sports Centre
- Playing Field
- Racquet Sports Facility
- Rugby Facility
- Recreation Ground
- Skateboarding Facility
- Civilian Firing Facility
- Tenpin Bowling Facility
- Water Sports Facility
- Winter Sports Facility

Full details of the classification scheme and the types of facilities covered can be found via the link above.

3. Details of Sensitivity Analyses (results summarised in main text)

Adjustment for behavioural confounders (Supplementary Table 1)

Diet is a strong predictor of adiposity but inclusion of total energy intake as a covariate in PA environment-adiposity models would potentially induce selection bias through substantial sample size restriction, as well as confounding bias through other backdoor pathways (Fig 1a, main report) Therefore, we further adjusted PA environment models for dietary intake (using a continuous measure of total energy intake (KJ), based on 24-hour recall dietary assessment). For consistency, additional models of the food environment were adjusted for physical activity. PA was operationalised as self-reported total energy expenditure through physical activity, captured with the self-reported International Physical Activity Questionnaire (IPAQ), expressed in terms of metabolic equivalent (MET) minutes per week, calculated and then categorised (to overcome skewness) according to the IPAQ short form guidelines to reflect low, moderate or high levels of PA.

Sample restriction based on diet and PA data (Supplementary Table 1)

As dietary data were only collected from a subset of 42% of the sample, we also explored whether any effect size attenuation in models adjusted for diet was being driven by selection bias due to missing dietary data, rather than adjustment, by comparing results for the main PA environment models with results from the same model run using only the subsample with dietary data.

PA was missing for 9% of the sample, and for consistency we also ran the fast food environment model on the subsample with PA data, for comparison with the main food environment model.

This enabled us to assess whether any observed differences in estimates when adjusting for behaviours (diet or PA) were being driven by the adjustment (i.e. confounding is present) or by selection bias due to missing data.

Bioimpedance BMI only (Supplementary Table 2)

5580 participants had their BMI calculated from weight measurements taken using standard scales rather than the impedance machine. To test the sensitivity of our results to this, we also estimated models using a version of the BMI measure in which these observations were excluded. Results were almost identical to the primary models.

Sample sizes for the sensitivity analyses varied depending on data completeness for the specific outcome and covariates. Ns are shown in the tables.

Supplementary Table 1. Results from sensitivity analyses examining impact of adjusting PA environment models for diet and food environment models for physical activity

	Adjusted for diet			*Restricted to subsample with dietary data (but no adjustment for diet)*		
**Number of PA resources in 1km street network buffer**	**WC**	**BMI (kg/m2)**	**% body fat**	**WC**	**BMI (kg/m2)**	**% body fat**
	n=177,288	n=177,143	n=174,780	n=177,288	n=177,143	n=174,780
**0**	ref	ref	ref	ref	ref	ref
**1**	-0.21 (-0.44, 0.01)	-0.11 (-0.20, -0.02)	-0.20 (-0.31, -0.08)	-0.21 (-0.44, 0.01)	-0.11 (-0.20, -0.02)	-0.20 (-0.32, -0.08)
**2-3**	-0.47 (-0.75, -0.20)	-0.24 (-0.35, -0.13)	-0.32 (-0.48, -0.16)	-0.47 (-0.74, -0.20)	-0.24 (-0.35, -0.13)	-0.32 (-0.49, -0.16)
**4-5**	-0.82 (-1.18, -0.45)	-0.41 (-0.56, -0.27)	-0.60 (-0.79, -0.41)	-0.81 (-1.18, -0.45)	-0.41 (-0.56, -0.27)	-0.61 (-0.80, -0.41)
**6 or more**	-1.24 (-1.69, -0.79)	-0.57 (-0.74, -0.41)	-0.83 (-1.05, -0.60)	-1.24 (-1.69, -0.79)	-0.57 (-0.74, -0.41)	-0.83 (-1.05, -0.60)
	Adjusted for PA			*Restricted to subsample with PA data (but no adjustment for PA)*		
**Distance to nearest fast-food outlet (m)**	**WC**	**BMI (kg/m2)**	**% body fat**	**WC**	**BMI (kg/m2)**	**% body fat**
	n=373,624	n=373,286	n=368,181	n=373,624	n=373,286	n=368,181
**<500m**	ref	ref	ref	ref	ref	ref
**500-999m**	-0.18 (-0.33, -0.03)	-0.09 (-0.16, -0.03)	-0.09 (-0.17, -0.01)	-0.17 (-0.32, -0.02)	-0.09 (-0.15, -0.02)	-0.09 (-0.17, 0.00)
**1000-1999m**	-0.28 (-0.49, -0.06)	-0.12 (-0.22, -0.02)	-0.09 (-0.21, 0.02)	-0.24 (-0.46, -0.02)	-0.11 (-0.21, -0.01)	-0.07 (-0.19, 0.05)
**At least 2000m**	-0.31 (-0.54, -0.07)	-0.12 (-0.25, 0.02)	-0.13 (-0.27, 0.02)	-0.27 (-0.52, -0.01)	-0.10 (-0.24, 0.04)	-0.10 (-0.25, 0.05)

Supplementary Table 2. Results from sensitivity analyses excluding BMI measurements taken using standard scales rather than bioimpedance machine

	Impedance-only BMI
**Number of PA resources in 1km street network buffer**	**BMI (kg/m2)**
	n=395,855
**0**	ref
**1**	-0.08 (-0.15, 0.00)
**2-3**	-0.18 (-0.28, -0.07)
**4-5**	-0.33 (-0.47, -0.20)
**6 or more**	-0.56 (-0.73, -0.39)
**Distance to nearest fast-food outlet (m)**	n=395,855
**<500m**	ref
**500-999m**	-0.08 (-0.15, -0.02)
**1000-1999m**	-0.11 (-0.20, -0.01)
**At least 2000m**	-0.10 (-0.24, 0.04)

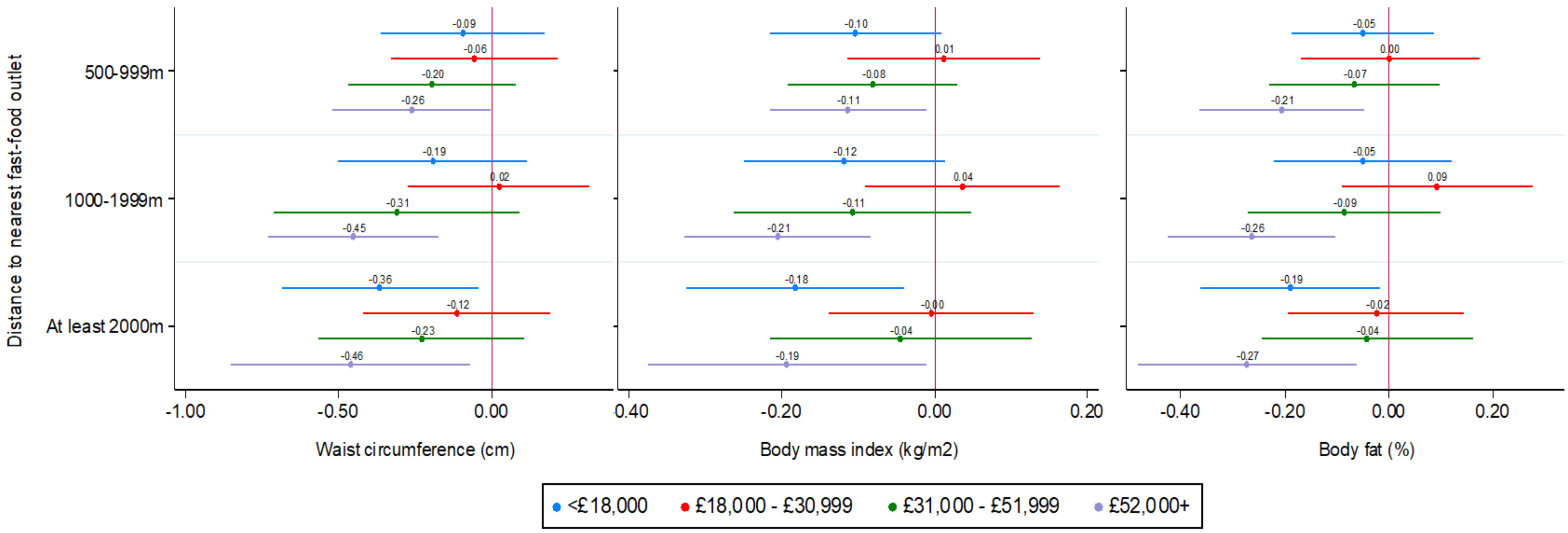

Supplementary Figure 2. Association between distance to nearest fast-food outlet and adiposity, by annual household income

Figure shows annual-household-income-stratified, fully adjusted mean differences in adiposity and associated 95% confidence intervals. The red line at zero represents the reference category (living <500m from nearest fast-food outlet).
